# Study on the Flexural Performance of Ultrahigh-Performance Concrete–Normal Concrete Composite Slabs

**DOI:** 10.3390/ma17184675

**Published:** 2024-09-23

**Authors:** Zizhou Sun, Xianjing Li, Chao Liu

**Affiliations:** 1 College of Civil Engineering, Tongji University, Shanghai 200092, China; szz150608@163.com; 2 Lin Tung-Yen & Li Guo-Hao Consultants Shanghai Co., Ltd., Shanghai 200437, China; lixianjing@shlinli.com

**Keywords:** UHPC–NC composite slab, bending test, parameter analysis, calculation formula

## Abstract

In recent years, there have been an increasing number of examples of using ultrahigh-performance concrete (UHPC) as a pavement layer to form an ultrahigh-performance concrete–normal concrete (UHPC–NC) composite structure to improve the bearing capacity of bridges. In order to study the flexural performance of this kind of structure, this research studied the flexural performance of UHPC–NC composite slabs, with UHPC in the compression zone, using experiments, numerical simulation, and theoretical analysis. The results showed the following. Firstly, after the UHPC–NC interface had been chiseled, there was no obvious slip between the two materials during the test, and the composite plate was always subjected to synergistic stress. Secondly, the composite slabs in the compression zone of the UHPC were all subjected to bending failure, and the cooperative working performance of each part under the bending load was good, indicating that the composite slab had a unique failure mode and a high bearing capacity. Thirdly, increasing the thickness of the UHPC significantly improved the flexural capacity of the composite plate, and the maximum increase was about 15%. Increasing the reinforcement ratio of the tensile steel rebars also had an increasing effect, with a maximum increase of about 181%. Finally, the proposed formula for calculating the flexural capacity of composite slabs with UHPC in the compression zone could accurately predict the bearing capacity of said slabs. The calculated results were in good agreement with the experimental values, and the error was small.

## 1. Introduction

Ultrahigh-performance concrete [1] (UHPC) is a new type of material. Steel fibers are evenly distributed in the matrix to make it have excellent mechanical properties and durability. The application of UHPC in bridge structures can significantly improve their bearing capacity and durability and prolong the service life of the bridge.

In recent years, much research has been carried out on the application of UHPC in bridge structures, both in China and abroad. Swiss scholars [2] first proposed the concept of an ultrahigh-performance concrete–normal concrete (UHPC–NC) composite structure, including UHPC–NC composite beams and composite slabs. In 2004, UHPC was applied to repair and reinforce concrete bridge decks in Switzerland, and related experimental studies were carried out. By the end of 2016, 93 bridges with UHPC as the main structure had been built in Malaysia, in which UHPC–NC composite beams accounted for 72% of the total structure [3]. In 2019, the China Railway First Academy tried, for the first time, to design a 56 m railway bridge solely supported by UHPC–NC composite beams, specifically in the 18th Lipu Bridge of the Lanzhou–Zhangjiakou high-speed railway. Finally, the weight of these composite beams is about 72% that of ordinary concrete [4].

For structures formed from UHPC–NC combinations, various scholars have carried out experimental research. Habel [5] believed that the use of UHPC improved the stiffness and ultimate bearing capacity of UHPC–NC composites, and the high density and tensile strain strengthening characteristics also improved the durability of the components. The addition of steel rebars to the UHPC layer could further improve the strength of the components, and three structural forms were proposed. Zanuy [6] further improved Habel‘s model, increasing the crack stress transfer mechanism and the interaction between the steel–concrete and concrete–UHPC interfaces. The stress transfer mechanism between adjacent cracks considered the characteristics of the material. Hor et al. [7] carried out an experimental study on UHPC-reinforced concrete slabs in the tensile zone. The results showed that the UHPC reinforcement layer could improve the overall stiffness of the slab and delay the development of shear cracks. Hou et al. [8] considered the influence of the UHPC layer’s depth and steel corrosion rate, deeply studied the flexural performance of UHPC-repaired corroded reinforced concrete beams, and proposed an analysis model for the flexural capacity of the repaired beams. Liao [9] studied the bonding strength of the UHPC–NC interface, found that UHPC could be used as a good bonding material, and proposed a method to improve the bonding strength. Prem et al. [10] studied the influence of the UHPC layer with different thicknesses on the ultimate flexural capacity of rectangular reinforced concrete beams when located in the tensile zone of the beams. With the change in thickness, the flexural capacity was greatly improved. Liu Chao et al. [11] carried out a bending performance test of three UHPC–NC beams and one ordinary reinforced concrete beam. The test results showed that the UHPC–NC composite beam essentially conformed to the assumption of a plane section during the loading process, and the UHPC layer was equipped with an appropriate amount of longitudinal reinforcement, which greatly improved the flexural capacity of the composite beam. Zingaila et al. [12] carried out a test and a numerical analysis of UHPC–NC composite slabs. Compared with ordinary concrete slabs, composite slabs had some evident advantages: the flexural capacity and stiffness were enhanced, while the crack width, spacing, and tensile stress of the steel rebars were reduced. Li Zhao et al. [13] studied the flexural performance of UHPC–NC composite beams and concluded that this type of beam can meet the requirements of flexural capacity under a normal service limit state and bearing capacity limit state. Yan et al. [14] carried out a four-point bending test of UHPC–NC composite slabs. The results showed that the composite slabs exhibited typical bending failure and had greater crack resistance, bending stiffness, and bending capacity than traditional concrete components. Deng et al. [15] carried out an experimental study on the flexural behavior of UHPC-reinforced beams of ordinary concrete. The results showed that UHPC reinforcement effectively improved the bearing capacity of the beams. Zhang Yang et al. [16] studied the flexural behavior of reinforced NC beams strengthened with UHPC. The UHPC reinforcement layer reduced the stress level of the tensile steel rebars and delayed the formation and development of cracks. Paschalis et al. [17] carried out a four-point loading test on reinforced concrete beams strengthened with UHPC in the tensile zone. The test results showed that the UHPC layer arranged on the tensile side of the reinforced concrete beam increased the stiffness of the beam and delayed the formation of cracks. Ganesh et al. [18] studied the fatigue performance of damaged reinforced concrete beams strengthened with a UHPC layer. The test results showed that UHPC reinforcement layers with different thicknesses improved the fatigue life of damaged beams. Tanarslan et al. [19] used UHPC to reinforce NC beams and tested the reinforcement effect with a four-point loading test. The results showed that the bearing capacity of the reinforced components was greatly improved, and the final failure mode of the components also changed. Safdar et al. [20] studied the bending performance of composite beams formed by replacing concrete materials at specific points with UHPC materials, providing significant guidance for the creation of new composite beams.

**Figure 1:**
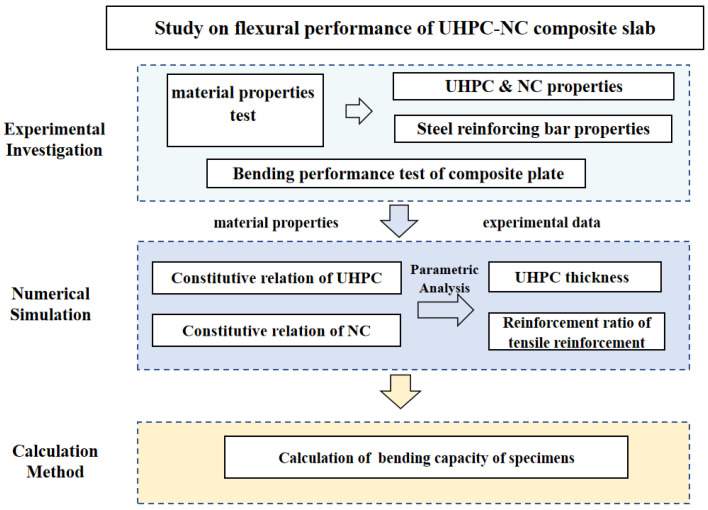
Research framework.

Compared with traditional concrete structures, UHPC–NC composite structures have good mechanical properties. Adding a UHPC layer on the top of existing concrete slabs has been proven to be an effective reinforcement method, such as in the Chillon Viaduct in Switzerland, which is reinforced by a layer of UHPC on the deck’s pavement [21]. At present, Chinese and other international scholars have carried out research on the bending performance of UHPC–NC composite structures, but there are few experimental studies on composite slabs in which the UHPC is in the compression zone. At the same time, when evaluating such structures under the current code, the default UHPC is all compressed, and the relationship between the thickness of the UHPC and the height of the compression zone is not considered, so that the bearing capacity of the composite slab cannot be accurately calculated. In this study, bending performance tests of multiple groups of slabs were designed and carried out, and a calculation formula for the bending capacity of composite slabs in which UHPC is in the compression zone was proposed. Figure 1 shows the research framework of this study.

## 2. Experimental Investigation

### 2.1. Material Properties

The materials and equipment used in this experiment were provided by Huaxin Chaokelong New Building Materials Technology (Huangshi) Co., Ltd. (Wuhan, Hubei Province, China) and Shanghai Swissdam Environmental Protection Technology Co., Ltd. (Shanghai, China). According to the relevant provisions of the GB/T 50152-2012 and T/CBMF 37-2018, various materials of the test slabs were tested. Table 1 and Table 2 provide the ratios and characteristic parameters of the steel fibers and the UHPC materials used in the specimens. Table 3 and Table 4 present the test results for the materials used in the specimens.

### 2.2. Design and Preparation of Specimens

**Figure 2:**
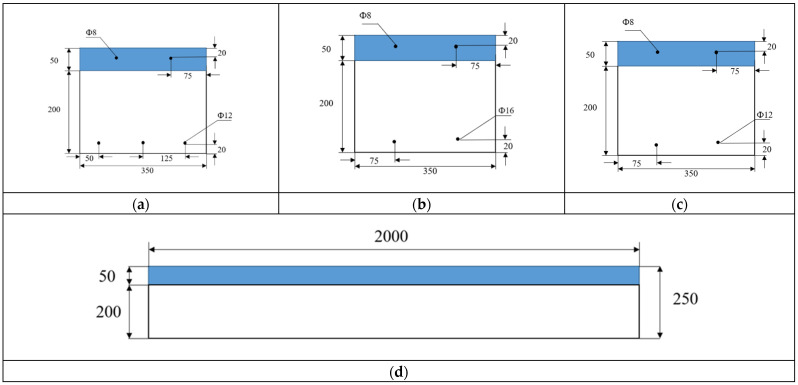
Dimensions of the specimens: (**a**) cross-section of B-1; (**b**) cross-section of B-2; (**c**) cross-section of B-3; and (**d**) longitudinal section of specimens. The blue shaded region represents the thickness of the UHPC, whereas the unshaded region represents the thickness of the concrete. All dimensions are in millimeters.

Three groups of UHPC–NC composite slab specimens numbered B1–B3 were designed. Each specimen was 2000 mm long, 350 mm wide, and 250 mm high. Among them, the UHPC’s height (h_u_) was 50 mm, and the concrete’s height (h_c_) was 200 mm. A schematic diagram and the specific parameters of the specimens are shown in Figure 2 and Table 5. The influence of the ratio of tensile reinforcement on the flexural performance of the test slab was investigated for different reinforcement layouts.

**Figure 3:**
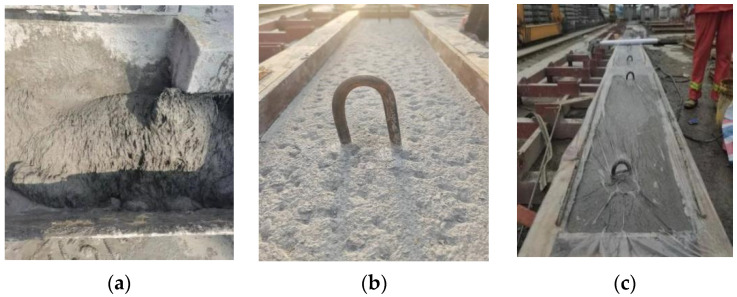
Preparation and casting of the specimens: (**a**) mixing the UHPC; (**b**) the chiseling treatment; and (**c**) curing the specimen.

According to the size of the specimens, a wooden mold was made; the lower layer of C50 concrete was first poured, and room-temperature moisturizing curing was maintained for 7 days. According to the literature [10,22,23], the UHPC–NC interface’s bonding performance is better under chiseling treatment, and no additional connection keys are required. Therefore, the interface was chiseled to remove the surface slurry and expose some hard aggregates. We cleaned the wet interface before pouring the UHPC so that the component was in a saturated wet state to increase the interfacial bond’s strength. After the UHPC had been poured, the film was cured for 48 h, and then, moisturizing curing was continued. After 28 days, the loading test was carried out. The process of making the specimens is shown in Figure 3.

### 2.3. Test Loading and Measurement Scheme

The experimental design was carried out with reference to the GB/T50152-2012. The bending test was carried out via the four-point loading method. The net span of the slab was 1800 mm, and the length of the pure bending section was 600 mm. The three specimens, namely B-1, B-2, and B-3, were subjected to positive bending moment loading; that is, the UHPC was located in the compressive layer and the ordinary concrete was located in the tensile layer.

**Figure 4:**
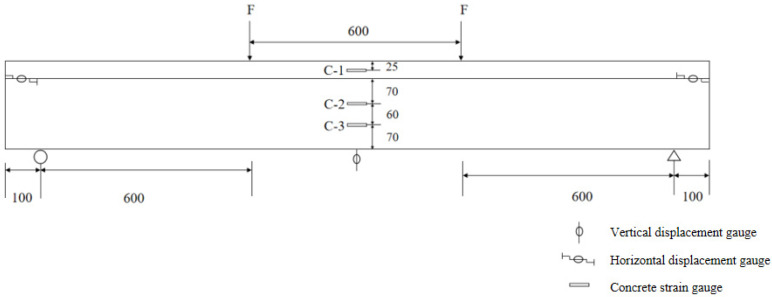
Diagram of the loading mode and the measuring points’ layout in the test; all dimensions are in millimeters.

**Figure 5:**
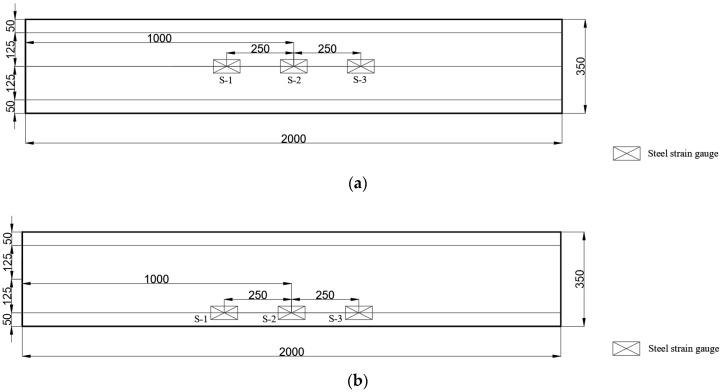
Layout diagram of the tensile steel strain gauge: (**a**) B-1 and B-2; and (**b**) B-3. All dimensions are in millimeters.

The vertical displacement meter and strain gauge were set in the middle part of the span. Two horizontal displacement meters were arranged at both ends of the interface between UHPC and ordinary concrete to see whether there was relative slipping between the two materials during the test. The strain gauges were arranged on the side of the specimen and were numbered C-1, C-2, and C-3 from top to bottom, as shown in Figure 4. The strain gauges were arranged at the tensile steel rebars and were numbered S-1, S-2, and S-3 from left to right, as shown in Figure 5.

**Figure 6:**
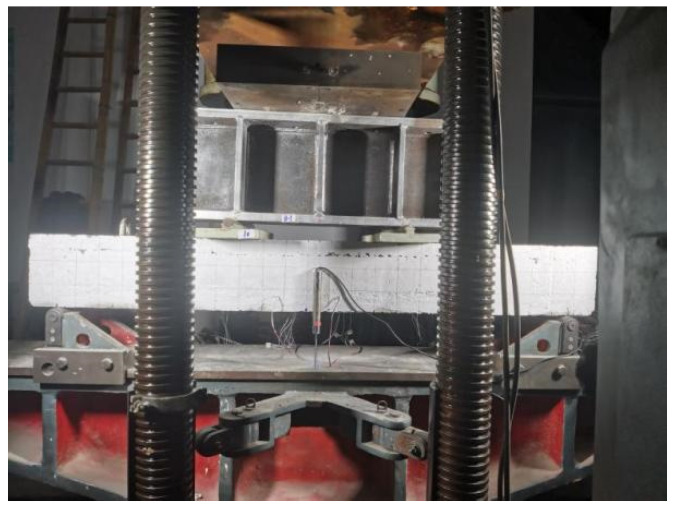
Setup of the loading test.

Before the test was officially loaded, it needed to be preloaded, and the maximum value of this load did not exceed 5% of the theoretical value of the failure load. During the formal loading, a load of 20 kN/level was applied to the component before the first crack was observed. When the first crack was observed, a 10 kN/level load was applied as the load. Under different loads, the data were collected for 5 min after reaching a stable state. The condition for stopping the test was when the bearing capacity of the specimen had reduced by at least 10% after reaching the limit, and the loading could be extended according to different failure phenomena. The process of loading the specimen is shown in Figure 6.

## 3. Experimental Results

### 3.1. Experimental Phenomena

**Figure 7:**
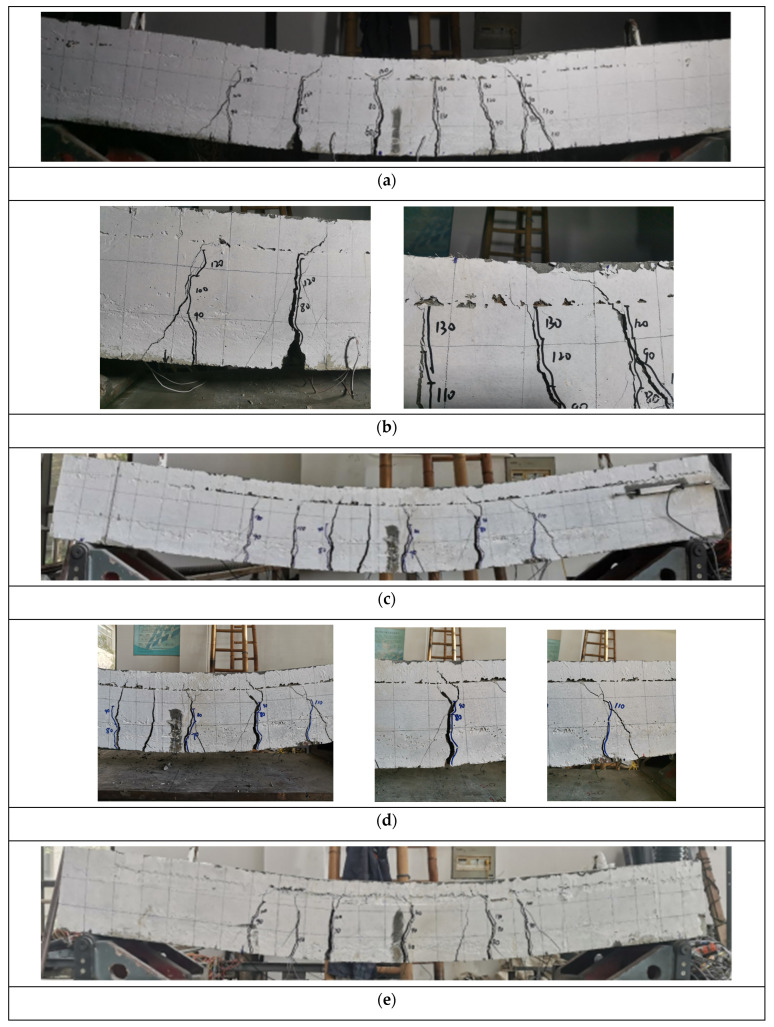

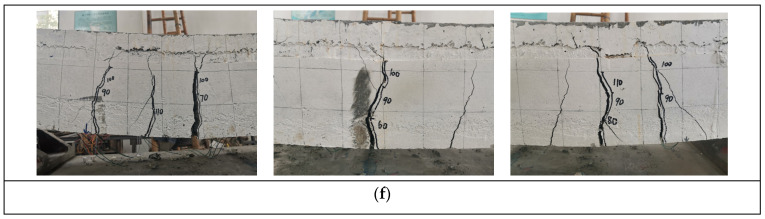
Cracking patterns in the test: (**a**) B-1; (**b**) enlarged images of the cracks in B-1; (**c**) B-2; (**d**) enlarged images of the cracks in B-2; and (**e**) B-3; (**f**) enlarged images of the cracks in B-3.

The ultimate load of the test slab and the displacement of the center of the bottom of the slab at the time of failure are shown in Table 6. In the early stage of loading, the mode of crack development in the specimen was similar to that of the ordinary concrete slab. However, when the crack developed to the interface between the UHPC and the concrete, it stayed in the vertical direction for a short time and then developed along the horizontal direction of the interface. However, according to the horizontal displacement meter at both ends, the UHPC–NC had good contact, and the displacement was only 0.01 mm, which can be considered as no horizontal relative displacement. The failure mode of the specimens is shown in Figure 7. The specific test phenomena were as follows.

(1) B-1: Under loading to 60 kN, the first crack appeared in the middle of the span. As the test continued, the load gradually increased, the number of cracks increased, and the cracks extended upward and gradually widened. When the specimen reached ultimate failure, the deformation of the specimen was obvious, and there were approximately eight cracks in the component. The cracks extended to the UHPC area, and the tensile steel rebars yielded. The specimen can be considered to have suitable reinforcement failure.
(2) B-2: When the specimen was loaded to 70 kN, the first crack appeared in the mid-span. As the test continued, the load gradually increased, the number of cracks increased, and the cracks extended upward and gradually widened. When the specimen reached ultimate failure, there were approximately eight cracks in the component. The cracks extended to the UHPC area, the tensile steel rebars yielded, and the deformation of the specimen was obvious. The specimen can be considered to have been damaged by reinforcement.
(3) B-3: Under loading to 70 kN, the first crack first appeared in the middle of the span. As the test continued, the load gradually increased, the number of cracks increased, and the cracks extended upward and gradually widened. When the specimen reached ultimate failure, there were approximately eight cracks in the component. The cracks extended to the UHPC area, the tensile steel rebars yielded, and the deformation of the specimen was obvious. The specimen can be considered to have been damaged by reinforcement.

According to the experimental phenomena, when the load was applied to a certain extent, the crack penetrated the lower concrete from the bottom to the interface between the UHPC and the concrete. While continuing to apply the load, the crack gradually extended to the UHPC layer. When the negative bending moment was loaded, cracks suddenly appeared in the UHPC layer and expanded rapidly.

Bending failure occurred in the three test slabs (B-1–B-3), and all of them were suitable for reinforcement failure; that is, the tensile longitudinal reinforcement yielded first, and the UHPC in the compression zone was crushed and destroyed. After the longitudinal reinforcement of the specimen yielded, the width of the main crack continued to grow, and the displacement continued to increase, resisting the external load. Finally, the UHPC in the compression zone reached the ultimate compressive strain and was crushed. Due to the high strength of the UHPC matrix, only the local area of concrete was crushed when it was destroyed.

### 3.2. Load–Displacement Curve

**Figure 8:**
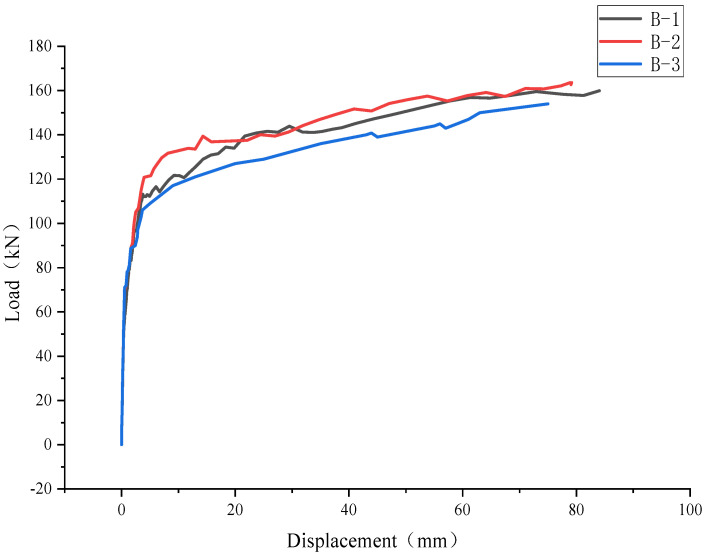
Load−displacement curves of composite slabs.

The load–displacement curve at the center of the bottom of each specimen was drawn from the deformation data collected in real time by the displacement meter fixed at the bottom of the slab, as shown in Figure 8. The change in the displacement of the composite slabs (B-1–B-3) under a positive bending moment was basically the same as when it was not cracked. When the load increased to about 110 kN, the change in the displacement of the composite slabs accelerated obviously until the ultimate load was reached.

### 3.3. Load–Strain Curve of Concrete

**Figure 9:**
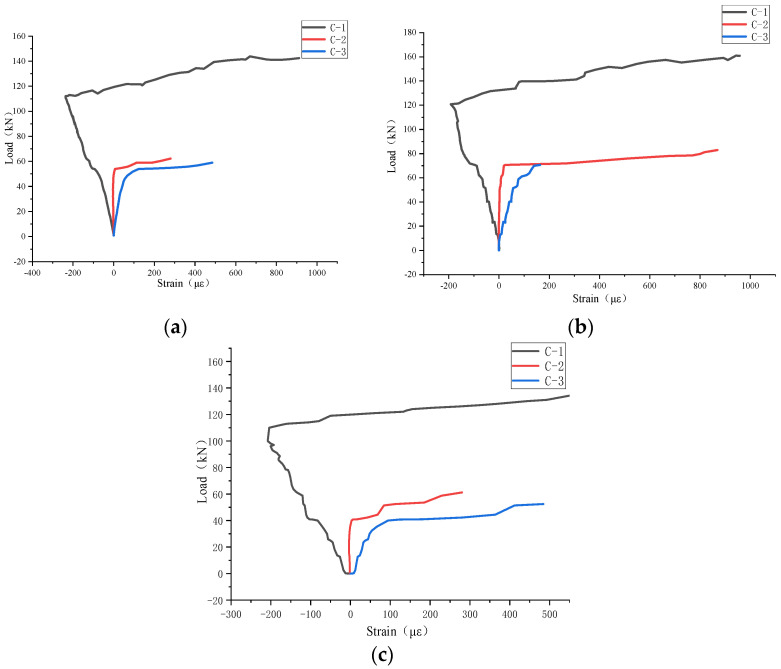
Load–strain curve of concrete: (**a**) B-1; (**b**) B-2; and (**c**) B-3.

From the strain data collected in real time by the strain gauge fixed on the slab side, the strain–load curves of each specimen and the load–displacement curves of the center of the bottom of each specimen were drawn, as shown in Figure 9. It can be seen from the figure that, at the initial stage of loading, Specimens B-1–B-3 were pulled at C-3 and pressed at C−1 and C−2. When the load of Specimen B-1 reached 50 kN and that of Specimen B-2 reached 60 kN, the strain gauges on the ordinary concrete (C-2, C-3) changed greatly; that is, the structure produced cracks. When the load continued to increase, the two strain gauges were damaged. The strain at the C-1 measuring point gradually increased until the load reached 110 kN, indicating that the UHPC had entered a stress state in which the upper side was under compression and the lower side was under tension until the final strain gauge was pulled out and the specimen reached the ultimate bearing capacity.

### 3.4. Load–Strain Curve of Tensile Steel Rebars

**Figure 10:**
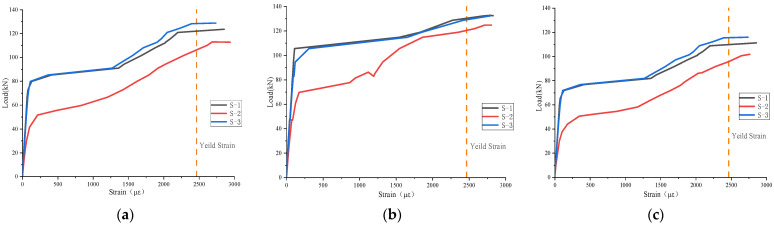
Load–strain curve of the tensile steel rebars: (**a**) B-1; (**b**) B-2; and (**c**) B-3.

The strain of the steel rebars in the mid-span section of the test beam is shown in Figure 10. The change in the strain of the steel rebars could also be divided into three stages. Before the specimen cracked, the strain of the steel rebars increased linearly. After cracking, the specimen entered the second stage. At this time, the tensile zone of the part entered the plastic stage, but the tensile longitudinal rebars were still in the elastic stage, and the strain increased linearly. However, after cracking, the tensile stress of the concrete/UHPC matrix partially transferred to the tensile longitudinal rebars and steel fibers in the crack, so the strain of the steel rebars increased rapidly, and the slope decreased slightly. After the steel rebars yielded, the specimen entered the third stage, when the steel rebars’ strain increased rapidly, and the strain gauge failed.

### 3.5. Distribution of Concrete Strain the in Mid-Span Section 

**Figure 11:**
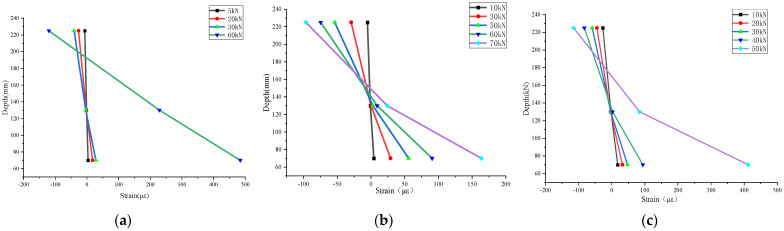
Concrete strain of the mid-span section of composite slabs: (**a**) B-1; (**b**) B-2; and (**c**) B-3.

The strain distribution of concrete in the mid-span section of the composite slab is shown in Figure 11. Figure 11 shows that the values of concrete strain for specimens B-1–B-3, from top to bottom, were 225 mm, 130 mm, and 70 mm from the bottom of the slab, respectively. When the load was small, the concrete strain was small, the concrete strain at different heights increased almost linearly, and the strain gauge could work normally. As the load increased, the neutral axis continued to move up, and after the section had cracked, the strain gauge exceeded its range and failed. When the test beam was close to the ultimate load, cracks developed to a large extent, and the strain gauge at the neutral axis basically failed. As seen in Figure 11, the strain in the mid-span section of the composite slabs remained approximately linear during the loading process; that is, each test slab satisfied the assumption of a plane section, providing a theoretical basis for the calculation of the bending capacity.

It can be seen from the above that B-1–B-3 were damaged by suitable reinforcement; that is, when the ultimate load had been reached, the tensile steel rebars yielded. Due to the high strength of the UHPC matrix, only the local UHPC was crushed during the failure. With an increase in the reinforcement ratio of the tensile steel rebars, the bearing capacity of the composite slab increased.

## 4. Finite Element Model

### 4.1. Basic Assumptions of the Materials’ Parameters

(1) Ordinary concrete

**Figure 12:**
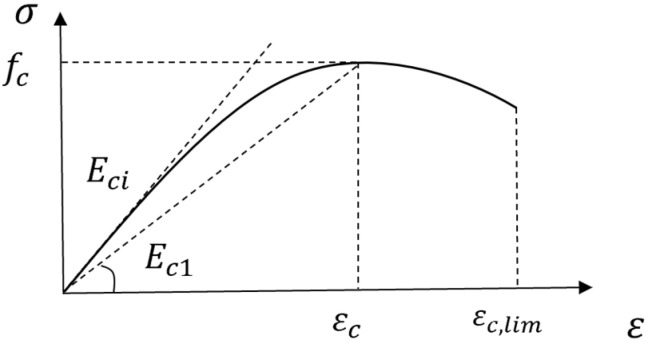
Constitutive relationship of concrete under compression in the finite element model.

The stress–strain relationship under uniaxial compression adopted the constitutive curve shown in Figure 12, and the tangent and secant modulus were used to define the curve shape of the rising and falling sections of the concrete. The parabolic constitutive equation is Equation (1), where 0.4 is the state of elastic compression.


(1) $$\sigma_{c}=-f_{c}\frac{k\eta -\eta^{2}}{1+(k-2)\eta }$$

The definitions of η and k are as follows:

(2) $$\eta =\varepsilon_{c}/\varepsilon_{c,1im}$$

(3) $$k=E_{ci}/E_{c1}$$

where $\varepsilon_{c}$ is the peak strain of the concrete, and $\varepsilon_{c,1im}$ is the ultimate strain of the concrete.

For C50 concrete, the value of $E_{ci}$ was 38.6 GPa, and the value of $E_{c1}$ was 23.2 Gpa.

**Figure 13:**
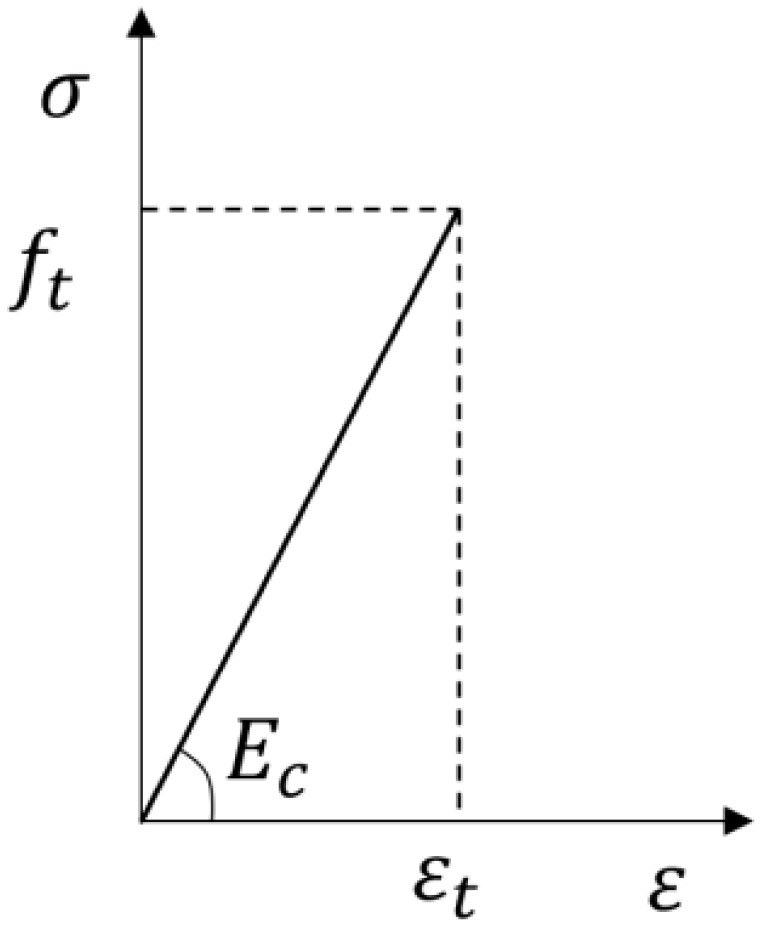
Constitutive relationship of the tensile performance of ordinary concrete.

In order to simplify the analytical model, the tensile performance of ordinary concrete only considered the elastic section, and its constitutive relationship is shown in Figure 13.

**Figure 14:**
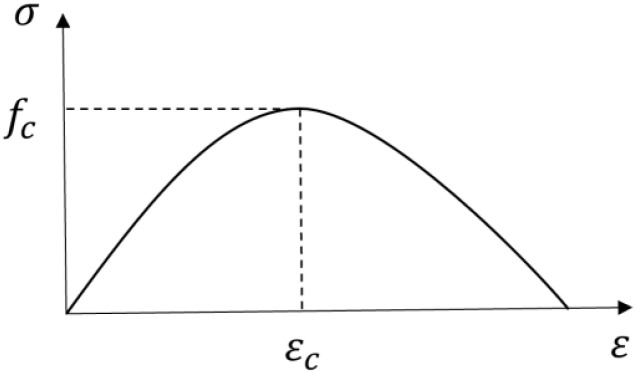
Constitutive relationship of the compression of ordinary concrete.

The compressive stress–strain curve of concrete adopted a simplified parabolic model. Its constitutive relationship is shown in Figure 14, and its expression is given in Equation (4):

(4) $$\sigma =f_{c}(\frac{2\varepsilon }{\varepsilon_{c}}-{\left(\frac{\varepsilon }{\varepsilon_{c}}\right)}^{2})$$

where ε is the compression strain of the concrete, and $\varepsilon_{c}$ is the peak strain of the concrete.

(2) UHPC

**Figure 15:**
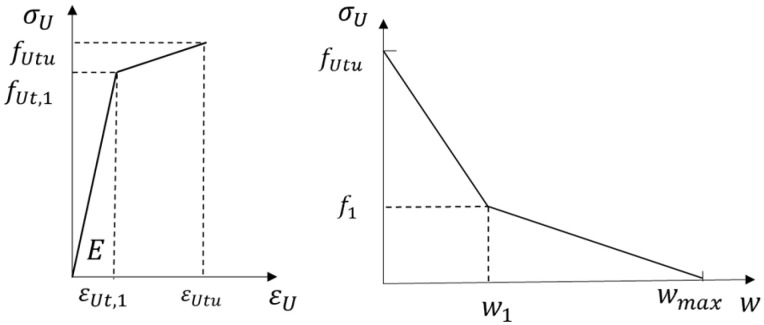
Tensile constitutive relationship of UHPC.

The tensile constitutive relationship of UHPC was a two-stage model, and the stress–strain curve before maximum stress was used. The tensile behavior after cracking was related to the cracks’ width; however, in order to facilitate the calculation of the whole analysis model, the stress–strain relationship of UHPC shown in Figure 15 was used, and its expression is shown in Equation (5).

(5) $$\sigma_{U}(\varepsilon )=\left\{\begin{array}{ll} f_{Utu}\left(\varepsilon_{1}-0.85\varepsilon -0.15\varepsilon_{Utu}\right)/\varepsilon_{1}-\varepsilon_{Utu} & \varepsilon_{Utu}\leq \varepsilon \leq \varepsilon_{1}\\ 0.15f_{Utu}(\varepsilon_{2}-\varepsilon )/(\varepsilon_{2}-\varepsilon_{1}) & \varepsilon_{1}\leq \varepsilon \leq \varepsilon_{2}\\ 0 & \varepsilon >\varepsilon_{2} \end{array}\right.$$

where $f_{Utu}$ is the peak tensile stress of UHPC; ε is the tensile strain of UHPC; $\varepsilon_{1}$ is the tensile strain corresponding to the turning point of the UHPC’s stress curve, with $\varepsilon_{1}=w_{1}/L_{eq}$; $\varepsilon_{2}$ is the ultimate tensile strain of UHPC, with $\varepsilon_{2}=w_{\max}/L_{eq}$; and $L_{eq}$ is the main crack’s spacing. Refer to reference [24] for the values.

**Figure 16:**
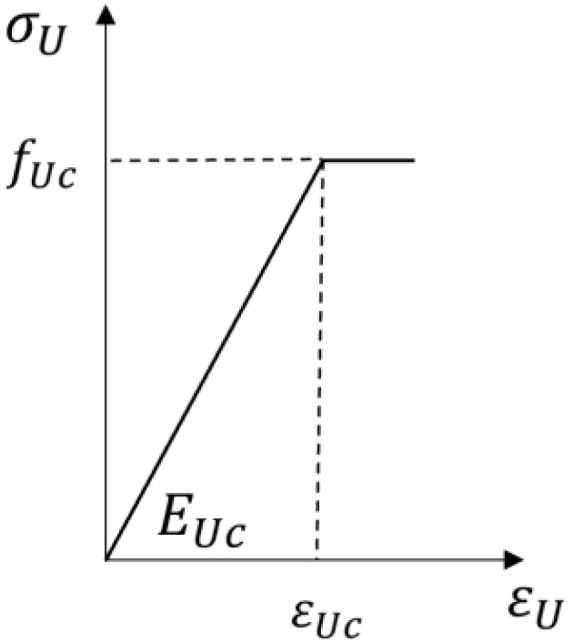
Constitutive relationship of the compression of UHPC.

The compressive stress–strain curve of UHPC adopted a bilinear model, which remained elastic before reaching the ultimate compressive strength, and then the stress remained unchanged. Its constitutive relationship is shown in Figure 16.

(6) $$\sigma_{U}=\left\{\begin{array}{ll} E_{Uc}\varepsilon_{U} & \varepsilon_{U}<\varepsilon_{Uc}\\ f_{Uc} & \varepsilon_{U}\geq \varepsilon_{Uc} \end{array}\right.$$

where $\varepsilon_{Uc}$ is the peak tensile strain of UHPC.

(3) Steel rebars

**Figure 17:**
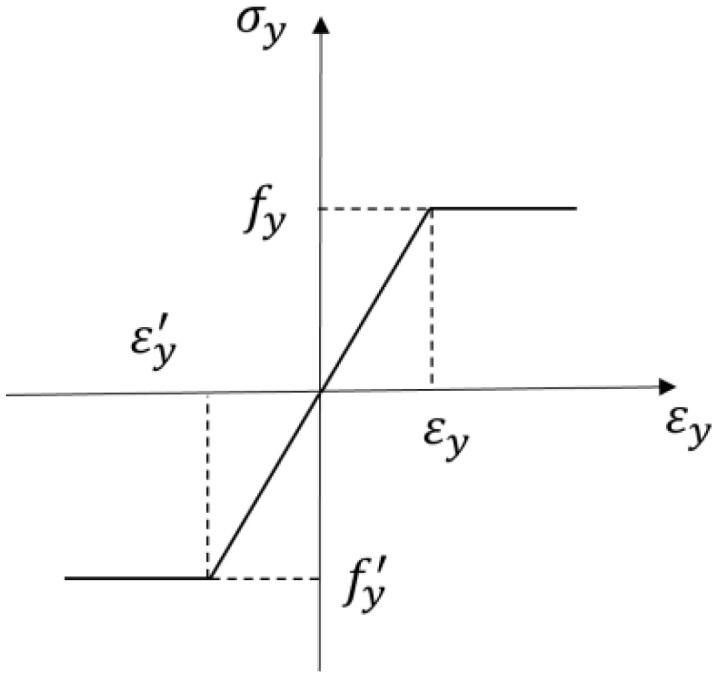
Constitutive relationship of steel.

Without considering the strengthening section of the steel rebars, the ideal elastic–plastic model was adopted, and the yield stress was assumed to be the maximum stress of the steel rebars. The steel rebars adopted the ideal elastic–plastic model, without considering the sections with strengthening and descending stress. Its constitutive relationship is shown in Figure 17.

(7) $$\sigma_{s}=\left\{\begin{array}{ll} E_{s}\varepsilon_{s} & \varepsilon_{s}<\varepsilon_{y}\\ f_{y} & \varepsilon_{s}\geq \varepsilon_{y} \end{array}\right.$$

where $\varepsilon_{y}$ is the peak tensile strain of UHPC, and $\varepsilon_{y}^{\prime }$ is the peak compression strain of UHPC.

### 4.2. Establishment of the Model

The specimens were numerically simulated using the finite element software ABAQUS 2020. In order to ensure consistency with the test site, models of the distribution beam and pad were established above the specimen. The boundary conditions of the specimen were simply supported on both sides, and the UHPC and ordinary concrete were bonded. The connection was typically selected according to the following principles when defining the main and secondary surfaces.

(1) The side with the larger material stiffness and structural stiffness was the main surface, and the side with the smaller material stiffness and structural stiffness was the secondary surface.
(2) The larger side was used as the main side, and the smaller side was used as the secondary side.
(3) The side with high roughness was the main surface, and the side with low roughness was the secondary surface. Since the NC side was greater than the UHPC part, the NC side of the contact surface was defined as the main surface, and the UHPC side was defined as the secondary surface. The steel rebars were embedded in the UHPC and ordinary concrete.

**Figure 18:**
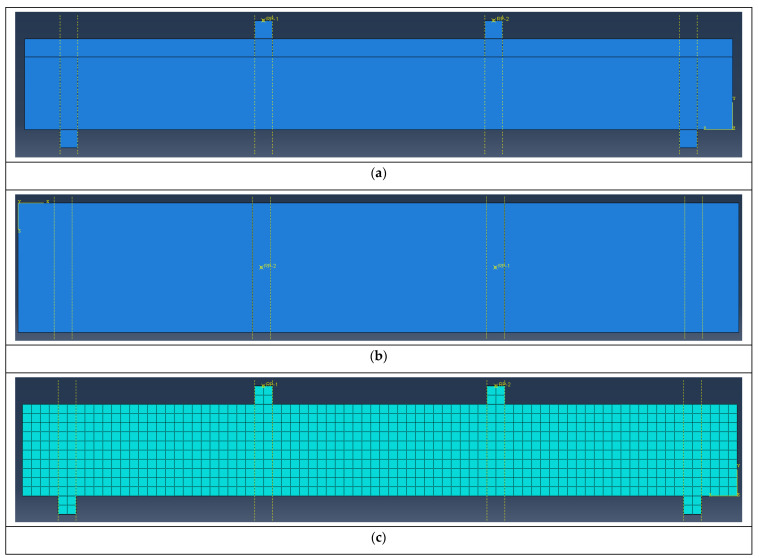
Diagram of the finite element model: (**a**) side view; (**b**) vertical view; and (**c**) subdivision of the mesh.

The mesh size was 25 mm, and the loading mode was displacement loading. The finite element model is shown in Figure 18.

### 4.3. Results of Numerical Simulation 

**Figure 19:**
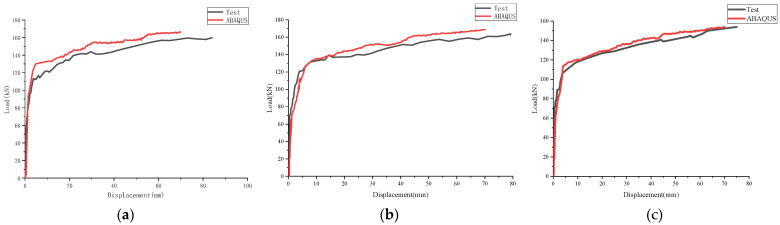
Comparison of experimental and numerical simulation data: (**a**) B-1; (**b**) B-2; and (**c**) B-3.

In the model described above, the vertical reaction force of the loading point and the maximum vertical displacement at the bottom of the mid-span were extracted, and the load–displacement curve of each specimen was obtained. The results of the finite element calculation and the test are shown in Figure 19 and Table 7. The trend of the load–displacement curve between the two was consistent overall, and the error of the ultimate bearing capacity was small. At the same time, the average and standard deviation were 0.97 and 0.01, respectively. This showed that the overall error between the results of finite element calculation and the test was small, and the ultimate load of the composite slab under the bending condition could be predicted more accurately.

### 4.4. Parametric Analysis

The test slab was calculated and compared using ABAQUS 2020. The results showed that the finite element model was more accurate. However, due to the limited number of test slabs, the UHPC’s thickness and the tensile reinforcement ratio were used as the parameter variables. The finite element model was used for parametric analysis. The parameters of each specimen in the analysis are shown in Table 8.

(1) UHPC thickness

**Figure 20:**
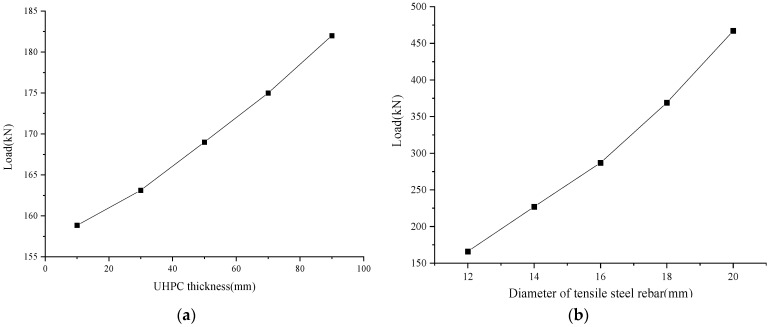
Analysis of the specimens’ parameters: (**a**) thickness of the UHPC; and (**b**) diameter of the tensile steel rebars.

In Table 8, B-U10 to B-U90 are the specimens used for the study of the thickness of the UHPC in the composite slabs. Maintaining other conditions consistent with B-1, a total of five thicknesses were set up for this series of specimens, which were 10 mm, 30 mm, 50 mm, 70 mm, and 90 mm. The results of the finite element model showed that, under bending conditions, the ultimate bearing capacity of the composite slab increased with an increase in the thickness of the UHPC layer and showed a certain linear relationship, as shown in Figure 20a. When the thickness of the UHPC layer increased from 10 mm to 90 mm, the ultimate load of the composite slab increased by about 15%.

(2) Tensile reinforcement ratio

In Table 8, B-S10 to B-S20 are the specimens used for the study of the tensile reinforcement ratio of the composite slab. In this series of specimens, while maintaining the other conditions consistent with B-1, five reinforcement ratios were set by adjusting the diameter of the steel rebars in the tensile zone. The diameters of the steel rebars were 12 mm, 14 mm, 16 mm, 18 mm, and 20 mm. The results of the finite element model showed that, with an increase in the reinforcement ratio in the tensile zone, the ultimate load of the composite slab also increased, and the growth trend gradually became faster. When the diameter Increased from 12 mm to 20 mm, the ultimate load increased by 181%, as shown in Figure 20b.

## 5. Theoretical Study on the Bending Failure of UHPC–NC Composite Slabs

The test results showed that the ultimate failure mode of the UHPC composite slab located in the compression zone was a typical reinforcement failure mode. Therefore, the flexural bearing capacity of the composite slab could be calculated according to the simplified plastic theory method.

When the UHPC is located in the compression zone, according to the relationship between the height of the compression zone ($x_{c}$) and the thickness of the UHPC layer ($h_{u}$), there are two cases for the distribution of stress in this section of the UHPC–NC composite slab as follows:

(1)
$x_{c}\leq h_{u}$

**Figure 21:**
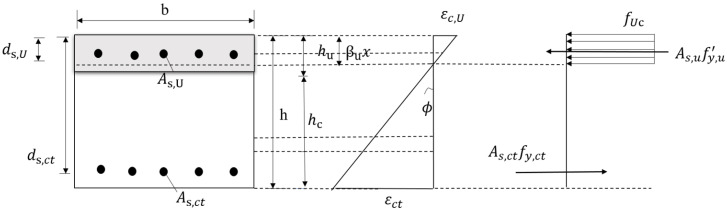
Simplified Model 1.

As shown in Figure 21, when the height of the compression zone was less than the thickness of the UHPC layer, the resulting force on the UHPC part of the compression zone could be expressed as follows:

(8) $$F_{U,c}=f_{Uc}b\beta_{u}x_{c}$$

where $f_{uc}$ is the compressive ultimate strength of UHPC, and $\beta_{u}$ is the coefficient of the reduction in height in the graph of stress in the UHPC in the compression zone, which was 0.75.

The balance equation for the axial force of the cross-section can be obtained according to the simplified model

(9) $$f_{Uc}b\beta_{u}x_{c}+f_{y,u}^{\prime }A_{s,U}=f_{y,ct}A_{s,ct}$$

where $f_{y,u}^{\prime }$ is the yield strength of the UHPC layer’s steel rebars, and $f_{y,ct}$ is the yield strength of reinforced concrete.

Taking the moment of the resulting force point of the tensile steel rebars of the ordinary concrete, the formula for the flexural capacity of the normal section of the UHPC–NC composite slab in the area of the positive bending moment is as follows:

(10) $$M_{u}=f_{Uc}b\beta_{u}x_{c}(d_{s,ct}-\frac{\beta_{u}x_{c}}{2})+f_{y,u}^{\prime }A_{s,U}(d_{s,ct}-d_{s,U})$$

Formula (10) needs to consider the yield of compressive steel rebars. If not, it is safe to ignore the contribution of the compressive steel rebars to bending. The formula for bending capacity is:

(11) $$M_{u}=f_{Uc}b\beta_{u}x_{c}(d_{s,ct}-\frac{\beta_{u}x_{c}}{2})$$

(2)
$x_{c}>h_{u}$

**Figure 22:**
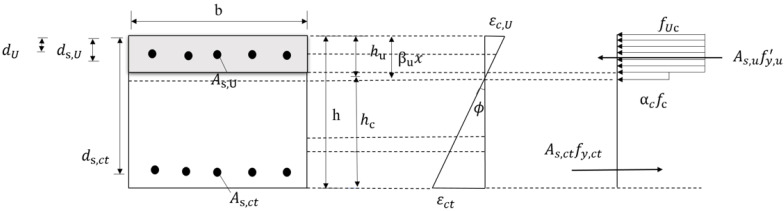
Simplified Model 2.

As shown in Figure 22, when the height of the compression zone was greater than the thickness of the UHPC layer, the resulting force of the UHPC part of the compression zone could be expressed as follows:

(12) $$F_{U,c}=f_{Uc}bh_{u}$$

According to the simplified diagram of the distribution of stress, the balance equation for the axial force of the section could be obtained:

(13) $$f_{Uc}bh_{u}+f_{y,u}^{\prime }A_{s,U}+f_{c}b(\beta_{u}x_{c}-h_{u})=f_{y,ct}A_{s,ct}$$

Taking the moment of the resulting force point of the tensile steel rebars of the ordinary concrete, the formula for the flexural capacity of the normal section of the UHPC–NC composite slab in the area of the positive bending moment is as follows:

(14) $$\begin{array}{l} M_{u}=f_{Uc}bh_{u}(d_{s,ct}-\frac{h_{u}}{2})+f_{y,u}^{\prime }A_{s,U}(d_{s,ct}-d_{s,U})\\ +f_{c}b(\beta_{u}x_{c}-h_{u})(d_{s,ct}-\frac{\beta_{u}x_{c}+h_{u}}{2}) \end{array}$$

Formula (14) needs to account for the yield of compressive steel rebars. If not, it is safe to ignore the contribution of the compressive steel rebars to bending. The formula for the bending capacity is as follows:

(15) $$M_{u}=f_{Uc}bh_{u}(d_{s,ct}-\frac{h_{u}}{2})+f_{c}b(\beta_{u}x_{c}-h_{u})(d_{s,ct}-\frac{\beta_{u}x_{c}+h_{u}}{2})$$

**Figure 23:**
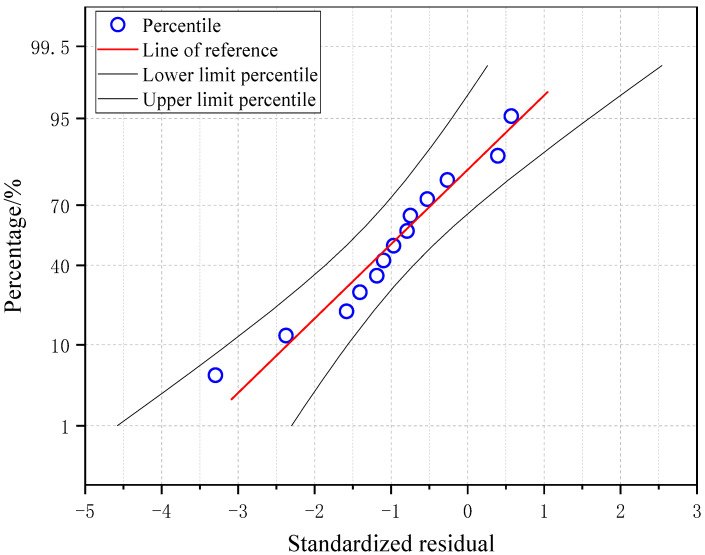
Standardized residual graph of the calculated results of the bending capacity.

In order to verify the correctness of the formula for flexural bearing capacity, the specimens in the test were calculated by the formula, and the results were compared with the measured values of the test slab. The results are listed in Table 9. It can be seen that the ratio of the measured value to the theoretical value fluctuated around 1.04. The average and standard deviation were 1.04 and 0.05, respectively. The overall dispersion of the calculation results was small and robust. In Figure 23, it can be seen that, except for individual points, the standardized residuals of the results of the flexural capacity formula were evenly distributed between −2 and +2, indicating that the flexural capacity formula could accurately calculate the flexural capacity of the specimen.

## 6. Conclusions

The bending test of three UHPC–NC composite slabs was completed, and a formula for the calculation of the flexural bearing capacity of the UHPC–NC composite slabs where UHPC was in the compression zone was proposed. The main conclusions are as follows:

(1) During the loading process, the bonding between UHPC and ordinary concrete was good, and there was no relative displacement at both ends. The two materials bore the load together as a whole. Therefore, the joint surface of the two met the requirements of interface bonding by chiseling.
(2) Bending failure of the composite slabs occurred, and all of the slabs involved the failure of the reinforced beam; that is, after the tensile longitudinal reinforcement had yielded first, some of the UHPC in the compression zone was crushed and destroyed. After the tensile steel rebars yielded, the width of the main crack continued to grow, and the displacement of the beam’s body continued to increase, resisting the external load. Finally, the UHPC in the compression zone was crushed and destroyed. Due to the high strength of the UHPC, only the local area of concrete was crushed when it was destroyed.
(3) The factors affecting the flexural capacity of composite slabs included the UHPC’s thickness and tensile reinforcement ratio. According to the results of this study, increasing the thickness of the UHPC significantly improved the flexural capacity of the composite slab, and the maximum increase was about 15%. Increasing the reinforcement ratio of tensile steel rebars also had an increasing effect, with a maximum increase of about 181%.
(4) A formula for calculating the flexural load capacity of the combined slabs where UHPC was in the compression zone was proposed, and the results of the calculation were in good agreement with the test values, with relatively small errors. Moreover, the formula could effectively calculate the flexural load capacity of the UHPC–NC combined slab.
(5) The size of the components in this test was limited by the test environment. In subsequent research, experiments could be conducted on on-site components in actual engineering to avoid the influence of size effects.
(6) The calculation formula proposed in this article was in good agreement with the experimental results. However, due to the limited sample size, these formulas are primarily intended as a reference in bridge design. Further experimental data are required for validation and refinement before they can be applied in the design of actual bridges.

## Figures and Tables

**Table 1:** Proportion of materials in the UHPC matrix (mass fraction).

Cement	Silica Fume	Fine Filler	Fine Aggregate	Water	Superplasticizer
1	0.176	0.311	1.400	0.222	0.027

**Table 2:** Characteristic parameters of steel fibers.

Fiber Shape	Tensile Strength (MPa)	Elastic Modulus (GPa)	Length (mm)	Diameter (μm)	Density (kg/m^3^)
Straight smooth	2875	201	14	200	7850

**Table 3:** Material properties of concrete and UHPC.

Material	Elastic Modulus E_c_ (GPa)	Compressive Strength f_c_ (MPa)	Tensile Strength f_t_ (MPa)
Concrete	34,500	43.6	2.8
UHPC	47,600	122.83	11.3

**Table 4:** Material properties of steel rebars.

Steel Grade	Elastic Modulus E_s_ (GPa)	Yield Strength f_y_ (MPa)	Ultimate Strength f_u_ (MPa)
HRB400	206,000	507.5	702.5

**Table 5:** Test parameters and details of the specimens.

Specimen	Material	Length (mm)	Width (mm)	Depth (mm)	UHPC Dimensions (mm)		NC Dimensions (mm)	
					Depth h_u_	Rebars	Depth h_c_	Rebars
B-1	UHPC and NC	2000	350	250	50	2Φ8	200	3Φ12
B-2	UHPC and NC	2000	350	250	50	2Φ8	200	2Φ16
B-3	UHPC and NC	2000	350	250	50	2Φ8	200	2Φ12

**Table 6:** Test results.

Specimen	Peak Load P_T_ (kN)	Central Displacement at P_T_ (mm)
B-1	161	85
B-2	165	78
B-3	150	72

**Table 7:** Comparison of the ultimate load between the test and numerical simulation.

Specimen	Test P_T_ (kN)	ABAQUS P_a_ (kN)	P_T_/P_a_
B-1	161	166	0.95
B-2	165	169	0.98
B-3	150	154	0.97
average			0.97
S.D.			0.01

**Table 8:** Parameters of the composite specimens.

Specimen	Length (mm)	Width (mm)	Depth (mm)	UHPC Depth h_u_ (mm)	NC Depth h_c_ (mm)	UHPC Rebars	NC Rebars	Tensile Reinforcement Ratio	Peak Load P_T_ (kN)
B-U10	2000	350	250	10	240	2Φ8	3Φ12	0.39%	159
B-U30	2000	350	250	30	220	2Φ8	3Φ12	0.39%	163
B-U50	2000	350	250	50	200	2Φ8	3Φ12	0.39%	169
B-U70	2000	350	250	70	180	2Φ8	3Φ12	0.39%	175
B-U90	2000	350	250	90	160	2Φ8	3Φ12	0.39%	182
B-S12	2000	350	250	10	240	2Φ8	3Φ12	0.39%	166
B-S14	2000	350	250	30	220	2Φ8	3Φ14	0.53%	227
B-S16	2000	350	250	50	200	2Φ8	3Φ16	0.69%	287
B-S18	2000	350	250	70	180	2Φ8	3Φ18	0.87%	369
B-S20	2000	350	250	90	160	2Φ8	3Φ20	1.08%	467

**Table 9:** Results of calculating the bending capacity (units: kN·m).

Specimen	Test M_T_	Formula M_u_	M_T_/M_u_
B-1	48.3	47.1	1.03
B-2	49.5	46.3	1.07
B-3	45	42.5	1.06
B-U10	47.7	47.1	1.01
B-U30	48.9	47.1	1.04
B-U50	50.7	47.1	1.08
B-U70	52.5	47.1	1.11
B-U90	54.6	47.1	1.16
B-S12	49.8	47.1	1.06
B-S14	68.1	65.9	1.03
B-S16	86.1	87.4	0.99
B-S18	110.7	111.6	0.99
B-S20	140.1	138.4	1.01
Average			1.04
S.D.			0.05

## Data Availability

The original contributions presented in the study are included in the article, further inquiries can be directed to the corresponding author.
